# Diffuse bronchiolitis pattern on a computed tomography scan as a presentation of pulmonary tumor thrombotic microangiopathy: a case report

**DOI:** 10.1186/1752-1947-5-575

**Published:** 2011-12-12

**Authors:** Marcos Duarte Guimarães, Maria Fernanda Arruda Almeida, Brelinger, Paula Nicole Barbosa, Rubens Chojniak, Jefferson Luiz Gross

**Affiliations:** 1 Department of Imaging, Hospital AC Camargo, São Paulo/SP, Brazil; 2 Department of Thoracic Surgery, Hospital AC Camargo, São Paulo/SP, Brazil

## Abstract

**Introduction:**

Pulmonary tumor thrombotic microangiopathy is a rare complication of malignant diseases. The diagnosis is extremely difficult and is most often performed after death. Invariably, patients develop acute pulmonary hypertension causing right heart failure, shortness of breath and death in a few days. We describe the clinical and radiological findings of a patient who presented with this complication.

**Case presentation:**

A 28-year-old Caucasian woman with a previous history of pelvic tumor resection two months previously, suggestive of metastatic adenocarcinoma, presented with intense shortness of breath. A computed tomography scan showed signs of acute cor pulmonale and diffuse nodular opacities associated with a tree-in-bud pattern disseminated through her lungs, suggestive of bronchiolitis. Our patient's condition worsened and she underwent a surgical biopsy. Pathologic analysis of the biopsied specimens revealed pulmonary tumor thrombotic microangiopathy. Our patient's tumor evolved from a gastric origin (Krukenberg tumor). She underwent progressive clinical deterioration and died less than 24 hours after the biopsy. None of the cases described previously in the literature had diffuse centrilobular nodular opacities associated with a tree-in-bud pattern disseminated through the lungs, as in our case.

**Conclusion:**

Pulmonary tumor thrombotic microangiopathy should be considered in cancer patients with rapidly progressing dyspnea, chest computed tomography findings compatible with pulmonary hypertension and typical findings of inflammatory bronchiolitis.

## Introduction

Pulmonary tumor thrombotic microangiopathy (PTTM) is a rare complication of malignant diseases. The diagnosis is extremely difficult and is most often performed after death [1]. Invariably, patients develop acute pulmonary hypertension causing right heart failure, shortness of breath and death in a few days [2]. We describe the clinical and radiological findings of a patient who presented with this complication.

## Case presentation

**Figure 1:**
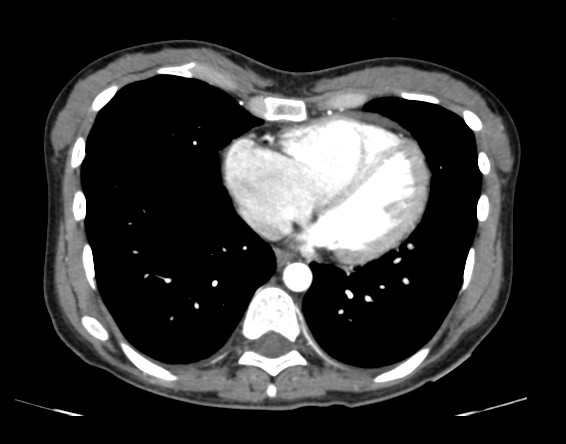
**CT axial series with medastinal window after infusion of intravenous contrast, showing increase in the size of the right atrium and right ventricle**. The right ventricular to left ventricular ratio is approximately 1.8.

**Figure 2:**
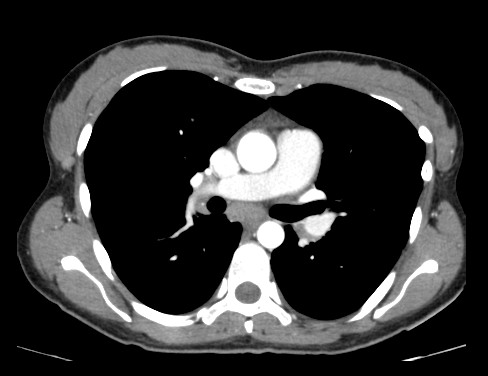
**CT axial series with medastinal window after infusion of intravenous contrast showing slight increase in the caliber of the pulmonary artery measuring about 30 mm**.

**Figure 3:**
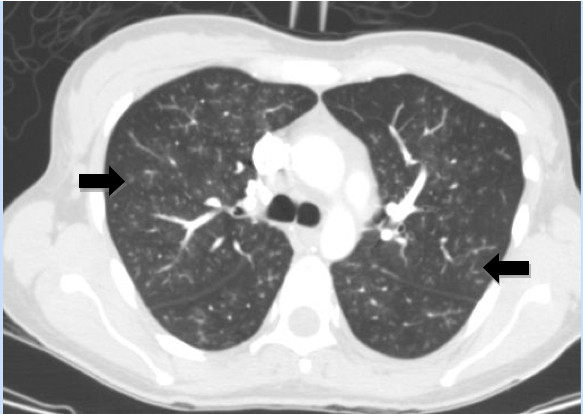
**High resolution CT scan shows diffuse pulmonary involvement with ill-defined centrilobular nodules and branching lines (arrows), which correspond to tree-in-bud appearance**.

**Figure 4:**
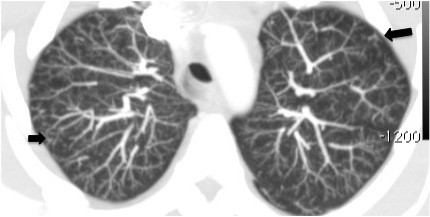
**CT axial series of the upper lobes with maximum intensity projection showing diffuse centrilobular nodular opacities with ground-glass attenuation associated with tree-in-bud pattern**.

**Figure 5:**
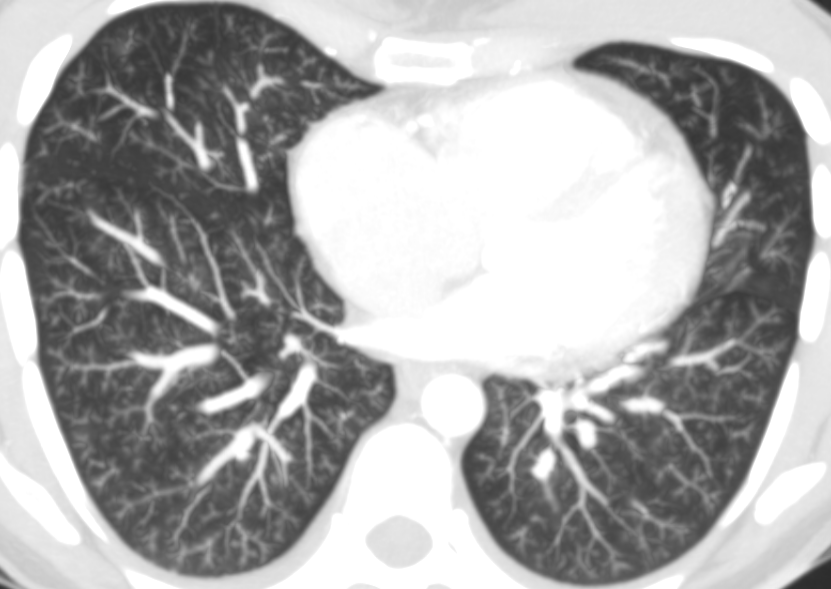
**CT axial series of the lower lobes with maximum intensity projection showing diffuse centrilobular nodular opacities with ground-glass attenuation associated with tree-in-bud pattern**.

**Figure 6:**
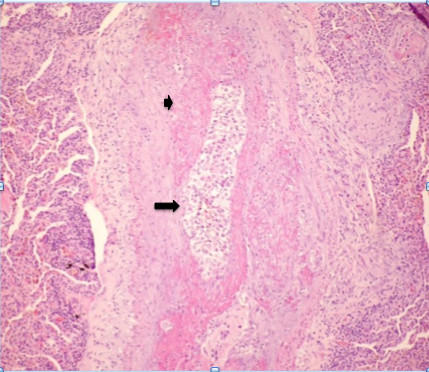
**Photomicrograph of histopathologic specimen shows complete arteriolar occlusion by tumor cells (arrow) and fibrointimal proliferation (small arrow), surrounded by lung tissue with collapsed alveoli**. (Original magnification ×400; hematoxylin-eosin stain.)

**Figure 7:**
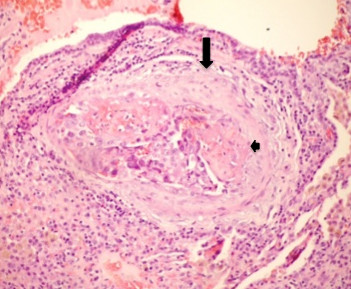
**Photomicrograph of histopathologic specimen shows fibrointimal proliferation (arrow) and presence of thrombi (small arrow) surrounded by tumor cells in centrilobular arteriole**. (Original magnification ×400; hematoxylin-eosin stain.)

A 28-year-old Caucasian woman presented to our hospital with a previous history of abnormal vaginal bleeding and abdominal pain that had begun four months prior to her initial presentation. A transvaginal ultrasound was performed at that time and demonstrated solid lesions in her left adnexal region, confirmed two weeks later with magnetic resonance imaging. She then underwent an exploratory laparotomy with a bilateral anexectomia. A pathological examination diagnosed a solid poorly differentiated adenocarcinoma, with signet ring cells diffusely infiltrating the ovaries and fallopian tubes bilaterally, compatible with a metastatic pelvic tumor of probable gastric origin, traditionally known as a Krukenberg tumor. After surgery, during the staging and treatment planning, our patient presented to our Emergency Room complaining of dry cough and shortness of breath of recent onset, with intense and progressive worsening. She had no other complaints and no fever. On examination, she was in generally poor condition, malnourished, dehydrated, with tachycardia and dyspnea; pulmonary auscultation revealed decreased breath sounds and mild wheezing in both lungs. Her leukocyte count showed no signs of infection; a blood test revealed the presence of mild anemia and a chest X-ray showed no abnormalities. Due to clinical suspicion of a pulmonary thromboembolism, our patient underwent a computed tomography (CT) scan of her chest, pelvis and thighs. Although the angiographic phase did not present any signs of pulmonary thromboembolism, the CT scan showed signs of acute cor pulmonale characterized by increased right heart chamber dimensions, slight interventricular septum deviation and a right ventricular to left ventricular ratio greater than 1.5 (Figures 1 and 2). In the lung window, the CT scan showed diffuse centrilobular nodular opacities with ground-glass attenuation, associated with a tree-in-bud pattern disseminated through her lungs, suggestive of bronchiolitis (Figures 3, 4 and 5). Our patient's condition worsened and, due to diagnostic uncertainty, she underwent a surgical biopsy with a wedge resection of her lung parenchyma. A right posterolateral thoracotomy was performed. Our patient's condition evolved with progressive clinical deterioration and she died less than 24 hours after the biopsy. Pathologic analysis of the biopsy specimens revealed pulmonary tumor thrombotic microangiopathy (Figures 6 and 7).

Laboratory investigations were remarkable for microcytic anemia (hemoglobin 11.2 g/dL, mean corpuscular volume 77 fL) and a strongly positive D dimer (1100 ng/mL). Her arterial blood gas examination breathing room air showed hypoxia with respiratory alkalosis (partial pressure of oxygen, 7.6 kPa; partial pressure of carbon dioxide, 3.9 kPa, pH 7.5). Our patient was admitted with a presumptive diagnosis of pulmonary thromboembolic disease.

## Discussion

PTTM is a rare complication of malignancies commonly detected after death. It was first described by von Herbay and colleagues as a diffuse intimal myofibroblast proliferation that causes pulmonary hypertension, leading to cardiopulmonary failure and death [3]. This disorder is found in a variety of malignancies, most often related to adenocarcinomas, especially of gastric origin [4,5]. Clinically, pulmonary tumor thrombotic microangiopathy manifests with signs and symptoms of pulmonary hypertension, that include progressive dyspnea, cough and hypoxia, also present in our patient. A cardiac evaluation may find tachycardia and signs of right heart failure on electrocardiography and on echocardiography. The patient develops rapidly progressive and fatal pulmonary hypertension. The clinical condition is often dramatic and usually the patient dies before diagnostic confirmation and treatment [6]. In our review, we found only one case reported in the literature of treatment success and patient survival [7].

Distinguishing between the clinical presentation of lymphatic and microvascular disease can be challenging, and many authors do not distinguish between these two. Soares and colleagues evaluated a series of 222 patients who had undergone autopsy, including 19 with microvascular pulmonary arterial embolism and 44 with lymphangitic carcinomatosis [8]. Although the tumor embolism group was more likely to have dyspnea (58%) than those with lymphatic disease (46%), the diagnostic utility of this symptom was low. Furthermore, a greater number of the tumor embolism patients had right ventricular enlargement than patients in whom lymphatic cancer predominated.

On histopathological investigation, pulmonary tumor thrombotic microangiopathy is characterized by widespread fibrocellular intimal hyperplasia of small pulmonary arteries, also called carcinomatous endarteritis induced by tumor microemboli [9]. Diffuse vascular occlusion in patients with pulmonary tumor thrombotic microangiopathy results in increased pulmonary vascular resistance [10]. Diffuse centrilobular nodular and a tree-in-bud pattern on thin-section CT is most often seen in patients with infectious bronchiolitis. This pattern is caused by the dilatation and plugging of small airways by mucus and inflammatory material and has been previously reported in some cases of pulmonary thromboembolism in the literature [11,12]. In our patient, there was extensive pulmonary thromboembolism of small arterioles with diffuse fibrointimal hyperplasia. Thus, the tree-in-bud pattern on thin-section CT, although usually caused by diseases of the small airways, may also be caused by a variety of vascular abnormalities, including pulmonary tumor embolism and pulmonary tumor thrombotic microangiopathy.

None of the cases described previously in the literature had diffuse centrilobular nodular opacities associated with a tree-in-bud pattern disseminated through the lungs, as in our case.

## Conclusion

PTTM should be considered in cancer patients with a rapidly progressing dyspnea, chest CT compatible with pulmonary hypertension and typical findings of inflammatory bronchiolitis.

## Consent

Written informed consent was obtained from our patient's next of kin for publication of this case report and any accompanying images. A copy of the written consent is available for review by the Editor-in-Chief of this journal.

## Competing interests

The authors declare that they have no competing interests.

## Authors' contributions

All the authors contributed to the writing of the paper. The original manuscript was written by MFA and AB, and the final version by MDG. RC and JLG contributed the CT images and additional references. All authors read and approved the final manuscript
